# Pediatric Cardiac Function Staging and Management Recommendations

**DOI:** 10.1002/pdi3.70063

**Published:** 2026-06-27

**Authors:** Bo Pan, Ling Han, Jianxin Zhuang, Xing Shen, Chenggang Mao, Yao Lin, Jindou An, Huili Zhang, Chunhong Xie, Yanmin Zhang, Li Zhang, Ying Guo, Yanyan Xiao, Shiwei Yang, Lei Zhang, Yue Yuan, Ling Sun, Zhi Chen, Wenhong Ding, Yifei Li, Zipu Li, Jie Tian

**Affiliations:** ^1^ Department of Cardiology, National Clinical Key Cardiovascular Specialty Children's Hospital of Chongqing Medical University National Clinical Research Center for Children and Adolescents' Health and Diseases Ministry of Education Key Laboratory of Child Development and Disorders Key Laboratory of Children's Important Organ Development and Diseases of Chongqing Municipal Health Commission Chongqing China; ^2^ Department of Pediatric Cardiology Beijing Anzhen Hospital Capital Medical University Beijing China; ^3^ Department of Pediatrics Shandong Provincial Hospital Affiliated to Shandong First Medical University Jinan Shandong China; ^4^ Department of Pediatrics The Affiliated Hospital of Southwest Medical University Luzhou Gansu China; ^5^ Department of Pediatrics The Affiliated Hospital of Qingdao University Qingdao Shandong China; ^6^ Department of Cardiology Capital Center for Children's Health Capital Medical University Beijing China; ^7^ Department of Pediatrics The First Affiliated Hospital Henan Medical College Zhengzhou University Zhengzhou Henan China; ^8^ Pediatric Cardiac Surgery Centre Fuwai Hospital National Centre for Cardiovascular Diseases State Key Laboratory of Cardiovascular Disease Chinese Academy of Medical Sciences and Peking Union Medical College Beijing China; ^9^ Department of Cardiology Children's Hospital, Zhejiang University School of Medicine National Clinical Research Center for Child Health Hangzhou Zhejiang China; ^10^ Department of Cardiology Xi'an Children's Hospital Affiliated Children's Hospital of Xi'an JiaoTong University Xi'an Shanxi China; ^11^ Department of Cardiology Guangzhou Women and Children's Medical Center, Guangzhou Medical University Guangzhou Guangdong China; ^12^ Department of Cardiology Shanghai Children's Medical Center School of Medicine Shanghai Jiao Tong University Shanghai China; ^13^ Department of Cardiology Cardiac Center Beijing Children's Hospital, Capital Medical University Beijing China; ^14^ Department of Cardiology Children's Hospital of Nanjing Medical University Nanjing Jiangsu China; ^15^ Department of Cardiology Children's Hospital of Soochow University Suzhou Jiangsu China; ^16^ Department of Cardiology Hunan Children's Hospital Changsha Hunan China; ^17^ Key Laboratory of Birth Defects and Related Diseases of Women and Children of MOE, Department of Pediatrics West China Second University Hospital, Sichuan University Chengdu Sichuan China; ^18^ Heart Center Qingdao Women and Children's Hospital Qingdao Shandong China

**Keywords:** cardiac function, pediatric heart failure, staging

## Abstract

The global prevalence of heart failure in children continues to increase, accompanied by a growing disease burden. To advance early prevention and intervention by shifting the focus upstream in the disease process, the Cardiovascular Group of the Pediatrics Society of the Chinese Medical Association has introduced the concept of pediatric cardiac function staging applicable to all children. This framework categorizes pediatric cardiac function into three distinct stages: the stage of cardiac function sufficiency (Stage A), the stage of cardiac dysfunction (Stage B), and the stage of cardiac function failure (Stage C). Based on this staging system, the diagnostic criteria and management strategies for each stage are delineated, with the aim of establishing a comprehensive management pathway rooted in cardiac function staging. This approach seeks to further reduce the incidence and burden of pediatric heart failure, providing a theoretical foundation and practical guidance for achieving the goal of making pediatric heart failure preventable, manageable, and treatable.

## 1 Background

Heart failure (HF), according to the Global Burden of Disease database, saw a 26.95% increase in global prevalence in children between 1990 and 2021 [1]. It is projected that the prevalence of pediatric HF and its associated disease burden will continue to increase until 2050 [1]. HF has become a major medical issue seriously threatening children's health, imposing a heavy burden on both the society and families. However, HF is not inevitable, and substantial evidence suggests that the focus should be on proactive prevention rather than passive response [2].

To achieve early prevention and treatment of pediatric HF, the International Society for Heart and Lung Transplantation (ISHLT), referencing adult HF staging, first proposed the concept of pediatric HF staging as early as 2004 [3]. In 2024, the Collaborating Group of Heart Failure and Collaborating Group of Precise Diagnosis and Treatment of Cardiomyopathy, Subspecialty Group of Cardiology, Society of Pediatrics, Chinese Medical Association, jointly proposed the “Recommendations on Staging and Management of Heart Failure in Chinese Children” [4]. Subsequently, in 2025, ISHLT again revised and affirmed pediatric HF staging in its updated guidelines [5]. These staging systems generally divide pediatric HF into the at‐risk for HF stage (Stage A), pre‐HF stage (Stage B), symptomatic HF stage (Stage C), and advanced HF stage (Stage D).

From a disease perspective, the concept of pediatric HF staging moves the focus of prevention earlier to the at‐risk for HF stage (Stage A), where HF risk factors are present. However, traditional risk factors can only explain approximately 50% of HF risk [6]. The existence and diversity of nontraditional risk factors (e.g., exposure to environmental toxins, chronic nutritional deficiencies, certain viral infections, and psychosocial stress) render individual risk assessment for pediatric HF more complex [2]. Standardized assessment methods for these factors are still evolving, posing challenges for comprehensive risk evaluation, particularly in the large population of apparently normal children.

To address these challenges, our proposed pediatric cardiac function staging system offers a distinct and complementary approach to existing guidelines. Although current frameworks (Chinese Recommendations 2024 [4], ISHLT 2025 [5]) focus on children already identified as being at risk for or having HF, our system expands the scope to include all children, regardless of risk factor status. This shift from a “disease‐focused” to a “function‐focused” approach enables true primary prevention at the population level. By centering on cardiac function itself, we can detect early changes before traditional risk factors are identified or before structural heart disease becomes clinically apparent. To effectively address these challenges and enable timely implementation of dynamic cardiac health management for all children—moving the prevention focus even earlier than the at‐risk‐for HF stage—the Heart Failure Collaborative Group and the Precise Diagnosis and Treatment of Cardiomyopathy Collaborative Group of the Cardiovascular Group, Pediatrics Society of the Chinese Medical Association, proposed the concept of “pediatric cardiac function staging” from the perspective of the cardiac function itself. They developed management recommendations for each stage and attempted to construct a pediatric HF prevention and management system based on cardiac function staging. This system includes all children within the scope of cardiac health management and embodies the “prevention first, moving the focus earlier” strategy for pediatric HF.

## 2 Terminology Related to Pediatric Cardiac Function

### 2.1 Cardiac Function Sufficiency (Normal Cardiac Function)

Cardiac function sufficiency refers to intact cardiac pumping and filling functions. It is characterized by hemodynamic stability, absence of pathological cardiac remodeling, having no abnormal cardiac biomarkers, and having no symptoms or signs related to impaired cardiac function. This population includes normal healthy children and children with HF risk factors but currently normal cardiac function.

### 2.2 Cardiac Impairment

Cardiac impairment refers to decreased cardiac pumping and/or filling function due to structural and/or functional abnormalities of the heart. Cardiac impairment is a pathophysiological concept encompassing the continuum from compensation (cardiac dysfunction) to decompensation (heart failure) [7].

#### 2.2.1 Cardiac Dysfunction

Cardiac dysfunction represents the compensatory stage of cardiac insufficiency. Objective evidence of this stage can be detected through cardiac biomarkers, cardiac imaging, or invasive cardiac tests [8]. Its core features include the presence of any one of the following [4]: (1) pathological cardiac remodeling changes, such as cardiac chamber enlargement, myocardial hypertrophy, abnormal wall motion, cardiac fibrosis, etc.; (2) abnormal cardiac function including global or regional systolic dysfunction, abnormal strain, diastolic dysfunction, and/or elevated ventricular filling pressure, confirmed by noninvasive and/or invasive tests (e.g., echocardiography, cardiac magnetic resonance, or cardiac catheterization); (3) presence of HF risk factors accompanied by elevated cardiac biomarkers (e.g., elevated B‐type natriuretic peptide [BNP], N‐terminal pro B‐type natriuretic peptide [NT‐proBNP], or persistently elevated troponin), after excluding other causes for the biomarker elevation. This stage can also be termed “asymptomatic cardiac insufficiency.”

#### 2.2.2 Cardiac Function Failure (Heart Failure)

Cardiac function failure is the decompensated stage of cardiac insufficiency, that is, clinical HF. It results from various etiologies leading to further worsening of the cardiac load and/or failure of compensatory mechanisms due to underlying structural and/or functional abnormalities. This causes a significant decrease in cardiac pumping and/or filling function, whereby cardiac output cannot meet the body's metabolic demands, significantly affecting the heart and other organs throughout the body [9]. It is characterized by hemodynamic instability, featuring not only measurable impairment of cardiac structure and/or function but also the presence of obvious clinical symptoms and/or signs of HF.

## 3 Pediatric Cardiac Function Staging Criteria

Based on the above concepts related to cardiac function, it is recommended to divide pediatric cardiac function into the following three stages: stage of cardiac function sufficiency (Stage A), stage of cardiac dysfunction (Stage B), and stage of cardiac function failure (Stage C).

**Table:** 

Cardiac function stage	Criteria
Stage of cardiac function sufficiency (stage A) (Normal cardiac function)	Intact cardiac pumping and filling functions. Absence of HF symptoms or/and signs Absence of pathological cardiac remodeling No elevation of cardiac biomarkers
Stage of cardiac dysfunction (stage B)	Absence of HF symptoms or/and signs, but presence of at least one of the following: Pathological cardiac remodeling Reduced ventricular systolic function and/or diastolic dysfunction and/or increased filling pressure HF risk factors accompanied by persistently elevated cardiac biomarkers (other causes must be excluded)
Stage of cardiac function failure (stage C)	Decreased cardiac pumping or/and function, accompanied by HF symptoms and/or signs.

## 4 Management for Pediatric Cardiac Function Staging

### 4.1 Staged Management Aims, Recommendations and Principles

#### 4.1.1 Stage of Cardiac Function Sufficiency (Stage A)

**FIGURE 1:**
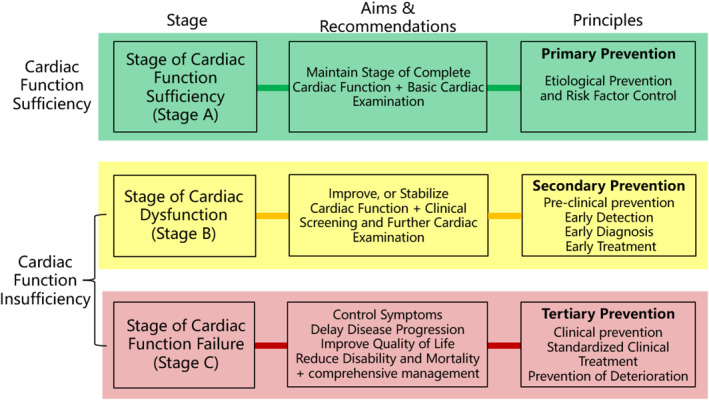
Principles, aims, and recommendations of pediatric cardiac function staging management. This flowchart outlines the core principles, aims, and key recommendations for managing each stage of pediatric cardiac function (A, B, and C). Stage A (normal cardiac function) focuses on primary prevention through cardiac health promotion, risk factor control, and selective screening. The goal is to maintain Stage A. Stage B (cardiac dysfunction) focuses on secondary prevention (“early detection, diagnosis, and treatment”) through etiological workup, targeted intervention, and close monitoring. The goal is to reverse to Stage A or stabilize in Stage B, preventing progression to C. Stage C (heart failure) focuses on tertiary prevention/clinical management through guideline‐directed therapy, multidisciplinary care, and advanced interventions. The goal is to control symptoms, improve prognosis, and potentially reverse to Stage B or A. Basic cardiac examination includes BNP/NT‐proBNP, 12‐lead electrocardiogram, and echocardiography. Clinical screening includes medical history, physical examination, and basic cardiac examination. Further cardiac examination includes speckle tracking echocardiography, three‐dimensional echocardiography, cardiac magnetic resonance imaging, cardiopulmonary exercise testing, 6‐min walk test, cardiac catheterization, myocardial biopsy, etc. BNP, elevated B‐type natriuretic peptide; NT‐proBNP, N‐terminal pro B‐type natriuretic peptide.

The focus of this stage is on primary prevention and control of HF risk factors for normal healthy children and children with HF risk factors but normal cardiac function (Figure 1). The core goals are to prevent, identify, and control potential HF risk factors. Management should be tailored considering the significant physiological differences across pediatric age groups (e.g., infants, school‐age children, and adolescents). Key strategies include the following:

1. Advocate for age‐appropriate cardiac health screening for all children and further assessment if necessary: For instance, incorporating cardiac evaluation (including history, physical exam, and basic cardiac examination as defined below) into regular child health checks is recommended. For children and adolescents engaging in competitive sports, systematic cardiac function assessments should be conducted before intense exercise, including consultation and examination by a pediatric cardiologist, 12‐lead electrocardiogram (ECG), echocardiography, and cardiopulmonary exercise testing (CPET) [10].
2. Provide education and implement control measures for potential HF risk factors. This includes establishing cohorts for children with a family history of conditions, such as diabetes, obesity, hyperlipidemia, hyperuricemia, coronary artery disease, congenital heart disease, or arrhythmia; developing reasonable early intervention measures; helping children establish good lifestyle and hygiene habits; and preventing the emergence of potential risk factors. Attention should also be paid to non‐conventional risk factors like environmental exposures and nutritional status.
3. Provide guidance on age‐appropriate diet and exercise management, promoting a balanced diet and effective weight control, which is particularly crucial for obese children [11].
4. For children with a family history of cardiomyopathy or known carriers of pathogenic genes, genetic counseling and genetic testing are strongly recommended, followed by regular clinical screening by a pediatric cardiologist. This screening should include detailed history, physical examination, 12‐lead ECG, echocardiography, and assessment of cardiac biomarkers [12].
5. Implement comprehensive interventions for other HF risk factors, such as hypertension, metabolic diseases, and congenital heart disease, without hemodynamic significance (e.g., small atrial septal defect).

Feasibility and Implementation Considerations for Stage A Screening:

Implementing cardiac screening for all children, as proposed in Stage A, requires consideration of feasibility and resource allocation. A tiered strategy is pragmatic and adaptable to varying resource settings. In high‐resource settings, comprehensive screening, including echocardiography, biomarkers (BNP/NT‐proBNP), and ECG, can be integrated into routine pediatric care and school‐based health programs. In medium‐resource settings, targeted screening focusing on high‐risk groups (e.g., children with a family history of cardiomyopathy, obesity, or congenital heart disease) using history and physical examination, and point‐of‐care ultrasound where available is recommended. In low‐resource settings, basic screening through risk stratification using simple questionnaires (e.g., family history and symptoms) and physical examination during routine visits can help identify children needing referral for further evaluation. This stratified approach maximizes early detection while optimizing healthcare resource utilization.

#### 4.1.2 Stage of Cardiac Dysfunction (Stage B)

The focus of this stage is on the early detection, diagnosis, and treatment of cardiac dysfunction from various causes. The core goals are to control, improve, or stabilize cardiac function, promote regression to Stage A, and prevent progression to Stage C. Key strategies include the following:

1. Conduct thorough etiological screening, encompassing family history, blood pressure measurement, metabolic profiles, immune markers, infection markers, and consideration of genetic testing.
2. Implement targeted etiological intervention, providing standardized diagnosis and treatments according to consensus guidelines for the underlying disease. Examples include surgical correction for congenital heart disease with hemodynamic significance and standardized anti‐inflammatory/immunotherapy for conditions such as myocarditis.
3. Strengthen and standardize the evaluation and monitoring of cardiac function. This involves systematic assessment of clinical symptoms and signs alongside relevant cardiac function tests. Essential tests include cardiac biomarkers (such as creatine kinase‐myocardial band, troponin I or T, and BNP/NT‐proBNP), echocardiography, and 12‐lead ECG. Optional or advanced tests should be prioritized based on the clinical scenario: cardiac magnetic resonance imaging (MRI) is particularly valuable when myocardial fibrosis, inflammation, or precise tissue characterization is suspected. CPET is highly recommended for functional capacity assessment, especially in older children and adolescents, and for evaluating exercise intolerance. Other tests such as speckle tracking echocardiography, three‐dimensional echocardiography, 6‐min walk test (for cooperative children), and cardiac catheterization (for clarifying hemodynamics or filling pressures), can be used as needed.
4. Develop individualized follow‐up plans based on the specific underlying disease and assessed risk level: For example, children with stable, low‐risk conditions (e.g., well‐controlled hypertension with normal echocardiography) may be followed every 6–12 months. Those with moderate‐risk or progressive conditions (e.g., certain cardiomyopathies and significant valvular disease) may require more frequent monitoring every 3–6 months. High‐risk patients (e.g., post‐myocarditis with residual dysfunction) might need follow‐up every 1–3 months initially.

#### 4.1.3 Stage of Cardiac Function Failure (Stage C)

The management principle for this stage is individualized, standardized clinical treatment. The core goals are to control symptoms, delay disease progression, improve quality of life, and reduce disability and mortality rates. Key strategies include the following:

1. Implementation of standardized guideline‐directed medical therapy (GDMT) for HF, adjusted for age and etiology.
2. Adoption of multidisciplinary team (MDT) collaborative management, integrating cardiac rehabilitation, nutritional support, and psychological care.
3. Assessment and rational application of non‐pharmacological therapies as indicated. These may include surgical repair or interventional therapy for underlying structural heart disease, cardiac resynchronization therapy (CRT), radiofrequency ablation (RFA), cardiac contractility modulation (CCM), implantable cardioverter‐defibrillator (ICD) implantation, extracorporeal membrane oxygenation (ECMO), and pulmonary artery banding (PAB).
4. Management of advanced HF, including assessment for indications for ventricular assist devices (VADs) and heart transplantation.

Following standardized treatment, children in this stage may achieve cure (regressing to Stage A), remission (regressing to Stage B), remain in Stage C requiring long‐term management, or experience further worsening of their conditions. The potential for reversal from Stage C to earlier stages is rooted in the concept of reverse cardiac remodeling. When the primary insult is removed or effectively treated (e.g., relief of volume overload, control of tachyarrhythmia, and resolution of inflammation), myocardial cells can recover, extracellular matrix remodeling may regress, and neurohormonal activation can be downregulated. This process is particularly robust in the pediatric population due to the ongoing maturation and greater plasticity of the developing heart. Early and aggressive intervention is crucial to maximize the opportunity for reverse remodeling before irreversible fibrosis occurs.

### 4.2 Integration With Existing Guidelines

The proposed pediatric cardiac function staging system is designed to complement and not replace existing pediatric HF guidelines (e.g., ISHLT 2025 [5] and Chinese Recommendations 2024 [4]). It offers three key innovations: (1) universal inclusion: expanding the Stage A population to all children, enabling early detection of cardiac dysfunction before traditional risk factors are identified; (2) function‐centered continuum: providing a simplified framework that tracks cardiac health from normality to failure, emphasizing the dynamic nature of cardiac function; and (3) pediatric‐specific reversibility: highlighting the unique potential for reverse remodeling in children, which supports more aggressive early intervention. Clinical decisions, especially in Stage C, should always be made in conjunction with detailed, etiology‐specific guideline recommendations.

### 4.3 Implementation Pathway for Cardiac Function Staging Management

**FIGURE 2:**
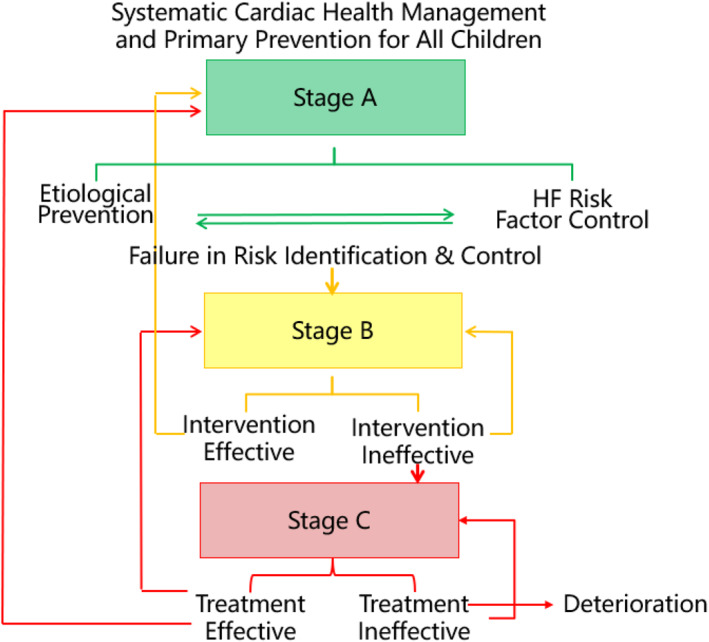
Implementation pathway for cardiac function staging management. This diagram illustrates the dynamic implementation pathway of pediatric cardiac function staging management. It starts with Stage A (all children), where primary prevention strategies are applied. Arrows indicate potential transitions: Effective risk factor control maintains Stage A, and uncontrolled risk factors or new insults can lead to progression to Stage B (cardiac dysfunction). In Stage B, successful early intervention can reverse function back to Stage A. Without effective intervention, progression to Stage C (HF) may occur. In Stage C, comprehensive treatment can lead to three outcomes: Reversal to Stage B (remission), reversal to Stage A (cure), or persistence in Stage C requiring long‐term management. The two‐way arrows between Stage A and Stage C highlight the potential for rapid progression or recovery unique to pediatrics. The core strategies for each stage (prevention, early Intervention, and clinical treatment) are shown in corresponding boxes. HF, heart failure.

Cardiac function staging management initiates at Stage A, involving primary prevention measures for all children, which encompasses systematic cardiac health management (Figure 2). During this process, it is essential to identify early various potential risk factors that may lead to cardiac dysfunction. Through the prevention, identification, regular follow‐up, and effective control of HF potential risk factors, the vast majority of the pediatric population is amenable to long‐term maintenance in Stage A.

If potential HF risk factors are not effectively identified and controlled, cardiac function may progress to Stage B. In Stage B, the core strategy of secondary prevention—“early detection, diagnosis, and treatment”—seeks to reverse cardiac function to Stage A or at least stabilize it in Stage B, thus preventing progression to Stage C.

Once Stage C is reached, comprehensive clinical treatment and prognosis improvement measures (tertiary prevention) are necessary to control the condition. The prognosis after treatment presents several possibilities: disease remission with regression to Stage B; significant improvement with reversal to Stage A; persistent condition requiring long‐term management, remaining in Stage C; or continued disease progression and worsening.

The spectrum of pediatric cardiovascular diseases differs significantly from that of adults [13]. Furthermore, the child's heart is still undergoing maturation and possesses a stronger capacity for reverse remodeling [14]. Consequently, in the pediatric population, cardiac function staging is more likely to exhibit direct progression from Stage A to Stage C (e.g., acute myocarditis in a previously healthy child) or direct reversal from Stage C to Stage A (e.g., after successful correction of a congenital heart defect) compared to the more gradual transitions often seen in adults.

### 4.4 Clinical Examples Illustrating the Stages

#### 4.4.1 Obesity

An obese adolescent with no cardiac symptoms, normal echocardiogram, and normal biomarkers is in Stage A. Management focuses on weight control and lifestyle modification. If obesity leads to pathological left ventricular hypertrophy on echo (even without symptoms), the child progresses to Stage B, requiring aggressive risk factor reversal and closer monitoring. If overt HF symptoms (e.g., exertional dyspnea) and systolic dysfunction develop, the child is in Stage C, requiring full HF management.

#### 4.4.2 Familial Dilated Cardiomyopathy (DCM)

A child with a pathogenic DCM gene variant but normal cardiac structure and function is in Stage A, requiring genetic counseling and periodic screening. If screening later reveals asymptomatic left ventricular enlargement and reduced ejection fraction, the child moves to Stage B, necessitating initiation of cardioprotective medications (e.g., angiotensin‐converting enzyme inhibitors or beta‐blockers). The development of symptoms such as fatigue and poor feeding would indicate Stage C.

#### 4.4.3 Congenital Heart Disease—Ventricular Septal Defect (VSD)

An infant with a small, restrictive VSD, normal growth, and no cardiac dysfunction is in Stage A, managed with observation. A large VSD causing significant left ventricular volume overload and chamber dilation but no overt HF symptoms places the infant in Stage B, prompting planning for surgical/interventional closure. If the same infant develops symptoms of congestive HF (tachypnea, poor feeding, or failure to thrive), they are in Stage C, requiring medical stabilization before definitive repair.

### 4.5 Challenge and Outlook

Pediatric heart failure represents a major public health challenge in China, particularly since treatment for children in Stage C is difficult and associated with high mortality. Against the backdrop of a continuously increasing disease burden, constructing the pediatric cardiac function staging system (Stages A/B/C) and implementing corresponding management strategies based on this staging are core pathways to achieving the goal of “preventable, controllable, and treatable” pediatric heart failure. This system shifts the focus of HF prevention and treatment earlier to encompass the Stage A and Stage B populations, addressing gaps in current guidelines regarding population‐level primary prevention. By strengthening primary and secondary prevention and achieving “early screening, early diagnosis, and early intervention,” the system is expected to significantly reduce the incidence of pediatric HF, delay disease progression, alleviate the overall disease burden, improve the efficiency of public health resource utilization, and comprehensively enhance the health quality of the pediatric population.

## Author Contributions

**Bo Pan:** conceptualization, methodology, writing – original draft, investigation, writing – review and editing, visualization. **Ling Han:** conceptualization, validation, visualization, writing – review and editing. **Jianxin Zhuang:** conceptualization, validation, visualization, writing – review and editing. **Xing Shen:** conceptualization, validation, visualization, writing – review and editing. **Chenggang Mao:** conceptualization, validation, visualization, writing – review and editing. **Yao Lin:** conceptualization, validation, visualization, writing – review and editing. **Jindou An:** conceptualization, validation, visualization, writing – review and editing. **Huili Zhang:** conceptualization, validation, visualization, writing – review and editing. **Chunhong Xie:** conceptualization, validation, visualization, writing – review and editing. **Yanmin Zhang:** conceptualization, validation, visualization, writing – review and editing. **Li Zhang:** conceptualization, validation, visualization, writing – review and editing. **Ying Guo:** conceptualization, validation, visualization, writing – review and editing. **Yanyan Xiao:** conceptualization, validation, visualization, writing – review and editing. **Shiwei Yang:** conceptualization, validation, visualization, writing – review and editing. **Lei Zhang:** conceptualization, validation, visualization, writing – review and editing. **Yue Yuan:** conceptualization, validation, visualization, writing – review and editing. **Ling Sun:** conceptualization, validation, visualization, writing – review and editing. **Zhi Chen:** conceptualization, validation, visualization, writing – review and editing. **Wenhong Ding:** conceptualization, validation, visualization, writing – review and editing. **Yifei Li:** conceptualization, validation, visualization, writing – review and editing. **Zipu Li:** supervision, conceptualization, methodology, writing – original draft, investigation, writing – review and editing, visualization. **Jie Tian:** supervision, funding acquisition, conceptualization, methodology, writing – original draft, investigation, writing – review and editing, visualization.

## Ethics Statement

The authors have nothing to report.

## Conflicts of Interest

Tian Jie is a Deputy Editor‐in‐Chief of *Pediatric Discovery*. To minimize bias, he was excluded from all editorial decision making related to the acceptance of this article for publication. The other authors declare no conflict of interest.

## Data Availability

Data sharing not applicable to this article as no datasets were generated or analysed during the current study.
